# Can Artificial Intelligence Help Orthopaedic Surgeons in the Conservative Management of Knee Osteoarthritis? A Consensus Analysis

**DOI:** 10.3390/jcm14030690

**Published:** 2025-01-22

**Authors:** Christian Carulli, Stefano Marco Paolo Rossi, Luca Magistrelli, Alessandro Annibaldi, Enzo Troncone

**Affiliations:** 1 Orthopaedic Clinic, University of Florence, Careggi University Hospital, 50121 Florence, Italy; 2 Department of Life Science, Health, and Health Professions, Università degli Studi Link, 00165 Rome, Italy; rossi.smp@gmail.com; 3 Sezione Chirurgia Protesica ad Indirizzo Robotico, Unità di Traumatologia dello Sport, Fondazione Poliambulanza, 25124 Brescia, Italy; 4 Ortopedia e Traumatologia, APUANE-NOA Hospital, 54100 Massa, Italy; luca.magistrelli@uslnordovest.toscana.it; 5 Institute of Sports Medicine and Science, CONI, 20137 Rome, Italy; alessandro.annibaldi91@gmail.com; 6 Butterfly Srl, 38123 Trento, Italy; enzo@butterflysrl.com

**Keywords:** knee osteoarthritis, consensus analysis, evidence-based medicine, artificial intelligence, decision support techniques, butterfly decisions

## Abstract

**Background:** Knee osteoarthritis is a prevalent condition that significantly impacts patients’ quality of life. Effective management typically involves a combination of pharmacological and non-pharmacological treatments. However, establishing a consensus on the optimal treatment strategy is crucial for standardizing care. The present study is the result of a rigorous process that combines artificial intelligence with human expertise to improve the reliability of medical recommendations. **Methods:** A new software platform (Butterfly Decisions, 2021, Italy) was employed to leverage AI-assisted decision-making, facilitating the digitalization of the entire consensus process. The process started with data collection through an online survey including simulated clinical cases of knee osteoarthritis collected by 30 orthopedic surgeons; artificial intelligence (AI) analyzed the collected clinical data and identified the key concepts and relevant patterns. Subsequently, AI generated detailed statements summarizing key concepts extracted from the data and proposed a reformulation of the statements to be discussed during the discussion session of the advisory board. The advisory board, composed of four qualified, experienced specialists of knee osteoarthritis, evaluated statements, providing their agreement levels, confidence, and supporting evidence. The AI tools calculated the degree of certainty and contradiction for each statement based on these evaluations. The literature was critically evaluated to ensure that there was an evidence-based evaluation of the proposed treatment statements. Finally, revised versions were proposed to address the feedback, evidence was collected to refine the scientific report, and the board members evaluated the AI performance too. **Results:** The consensus analysis revealed a high level of agreement in the need for a multimodal approach to treating knee osteoarthritis. The feedback highlighted the importance of integrating physical therapy and weight management, non-pharmacological methods, with Symptomatic Slow-Acting Drug for Osteoarthritis (SYSADOAs) and pharmacological treatments, such as anti-inflammatory drugs and intra-articular knee injections. The board members found that AI was easy to use and understand and each statement was structured clearly and concisely. **Conclusions:** The expert consensus about knee osteoarthritis conservative management being facilitated with AI met with unanimous agreement. AI-assisted decision-making was shown to have excellent analytical capabilities, but algorithms needs to be trained by orthopaedic experts with the correct inputs. Future additional efforts are still required to evaluate the incorporation of AI in clinical workflows.

## 1. Introduction

Knee osteoarthritis (KOA) is the most common joint disorder, affecting 300 million patients worldwide [1]. Symptoms consist of pain, effusion, a loss of function, and disability. The etiology of KOA is multifactorial, with risk factors including advanced age, genetic predisposition, previous knee injuries, and obesity. Despite the availability of various treatment options, managing KOA remains rather challenging due to its chronic nature and the variability in patient response to treatments [2].

With the introduction of artificial intelligence (AI) into medicine, there are new opportunities for data analysis that can be used for the decision-making process.

The importance of AI in medicine is increasing; currently, at least 30 AI medical devices and algorithms are approved by the US Food and Drug Administration in a variety of clinical disciplines. These tools are designed to operate with varying levels of autonomy and are rapidly becoming an integral part of healthcare [3]. Significantly, in 2020, the Centers for Medicare and Medicaid Services announced the first introduction of an AI platform to hospitals, which was a model used for the early detection of strokes [4].

In 2023, the American Medical Association conducted a survey for a comprehensive study of physician’s sentiments towards the use of AI in healthcare; in general, doctors are enthusiastic about the potential of AI in healthcare, with 65% seeing at least some advantage to the use of AI in their practice [5].

One application of AI models is in natural language processing (NLP), which involves various techniques such as text mining, sentiment analysis, and machine translation. NLP is an AI system that is able to create logical and contextually appropriate texts using learning algorithms. Once the program is trained, it can be used for a variety of tasks [6].

For example, it can be used as a predictive tool for mitigating the clinical and economic burden of different clinical challenges, like intraoperative periprosthetic fractures in total hip arthroplasty [7]; recently, there has been an increasing number of studies applying NLP algorithms in orthopedics [8,9,10,11,12,13]. In this field, several studies have already shown that NLP is a useful and reliable method; in contrast, a recent work revealed a limited correlation between responses generated by AI and expert statements on the diagnosis and treatment of anterior shoulder instability (ASI). In this study, Artamonov and colleagues evaluated the similarity of answers provided by Generative Pretrained Transformer-4 (GPT-4) with those of a consensus statement on the diagnosis and non-operative management of ASI [14].

These findings suggest that NLP methods are limited to the quality and quantity of data used to train them and healthcare professionals need to be adequately prepared to utilize these tools [15]. There are many potential clinical, research, and educational applications of large language models in orthopedics, but the development of these applications needs to be focused on patient safety and the maintenance of high standards. Consequently, the importance of the proper clinical validation of AI technologies used for medicine has recently been underscored by multiple high-impact peer-reviewed medical journals, and in the near future, the use of artificial intelligence in the EU will be regulated by the “AI Act”, the world’s first comprehensive AI law [16,17,18]. This regulation should apply from 2 August 2026 [19].

Many medical professionals are not familiar with the details of AI, its algorithms, or how it can be integrated into their practice, and another challenge is the integration of AI tools into the existing clinical workflow [20,21,22]. Only a minority of the studies integrate AI applications in clinical workflows and this is the first study that evaluates the incorporation of an AI-assisted decision-making process in KOA management. The consensus serves as a foundation for clinical decision-making. The authors think that an expert consensus promoting clinical excellence and the adoption of AI in this field may improve personalized healthcare enhancements.

## 2. Materials and Methods

The Butterfly Decisions platform (www.butterflydecisions.com/?lang=en, accessed on 16 December 2024) follows this approach to support medical decision-making [23]. An AI-assisted decision-making process, as described by Eric Colson (2020), combines advanced analytical capabilities of AI with expert human judgment to optimize decision outcomes [24].

### 2.1. Process Description

The consensus process was conducted using a software platform where participants evaluated specific treatment statements for KOA. The process began on the Butterfly Decisions platform, with the collection of 30 simulated clinical cases of mild to moderate knee arthritis via an online survey, from which key themes and concepts were then extracted for the formulation of statements through the use of context discovery algorithms. The identified statements were evaluated by an advisory board through a survey conducted on the Butterfly Decisions platform. The platform calculated and analyzed proprietary certainty and contradiction indices, as well as sentiment analysis, for each statement based on board members’ feedback. These insights, along with board members’ feedback and relevant literature, provided a foundation for AI prompts. These prompts were crafted using prompt engineering techniques, including role prompting, to optimize the integration of generative AI models within the system. Subsequently, these models reviewed and proposed a reformulation of the statements based on both advisory board feedback and supporting literature. Each phase of evaluation and voting was tracked on the platform with dynamic reports detailing the feedback and evidence provided. After the discussion and analysis phase by the board, the statements were further refined, before a final phase of online voting on the Butterfly Decisions platform. This second phase of voting, having reached full consensus, closed the process with the issuance of a final consensus report.

**Figure 1:**
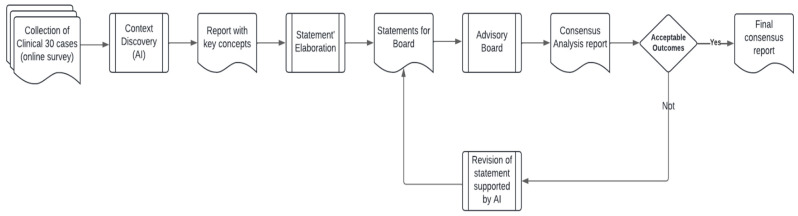
Process description of the study protocol.

A schematic overview of the study protocol is provided in Figure 1.

### 2.2. Collection of 30 Clinical Cases (Online Survey)

The process started with data collection through an online survey, including 30 simulated clinical cases of patients with symptomatic KOA. The questionnaire, presented in Italian, was structured around 4 main topics: symptoms, diagnosis, treatments, and outcomes. The survey was designed with a free-text option for detailed responses. Participation was entirely voluntary and anonymous, and data were deidentified; the participants were expert and dedicated orthopedic surgeons employed in Italian hospitals. The survey was created on a basis of 30 mild to moderate knee arthritis cases, described only by sentences related to symptoms or functional deficiencies referred to by several patients, but without any diagnostic images. All cases were intended to be managed in a conservative way, and the involved specialists were asked to answer the various issues by open free-text responses and comments.

### 2.3. Context Discovery (AI)

Context discovery used AI tools to analyze the collected data and identify key concepts and relevant patterns. This step aimed to understand the context of clinical data, isolating crucial information and trends that may influence medical decisions. AI performed an in-depth analysis of the data, extracting information that may not be immediately evident to humans.

### 2.4. Report with Key Concepts

Following AI analysis, based on natural language models and prompt engineering tools, a detailed report was generated, summarizing the key concepts extracted from the data. These data were the most common key points that arose from the various open answers and observations related to the 30 cases, as analyzed by AI. Graphs, tables, and textual explanations highlighting the main findings were thus obtained, hypothetically sparing time and efforts for a manual evaluation by physicians. It served as a reference document for the subsequent stages of the process, providing a clear and concise overview of the most relevant information. Based on the generated report, detailed statements were formulated, representing preliminary interpretations and recommendations. These statements were developed to provide specific clinical guidance, based on the evidence emerging from data analysis. Each statement was structured to be clear, concise, and easily understandable by the board members.

**Figure 2:**
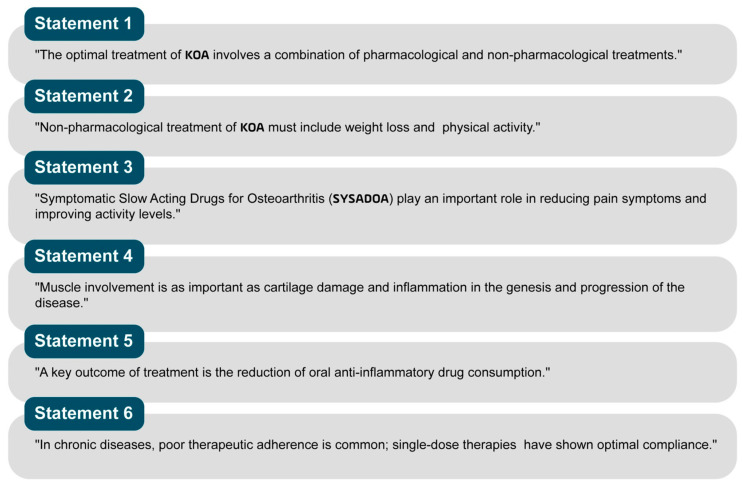
Statements.

The statements are shown in Figure 2.

### 2.5. Statement Elaboration

The consensus process was conducted using a software platform where participants evaluated specific treatment statements for KOA. Participants were asked to indicate their agreement or disagreement, provide a confidence rating, and supply supporting evidence. The platform calculated the degree of certainty and contradiction for each statement based on these evaluations. The statements were then analyzed, and revised versions were proposed to address the feedback and evidence collected.

### 2.6. Statement for the Board

The developed statements were then presented to the advisory board. This step was crucial to ensure that recommendations were accurate, practical, and clinically relevant. The board received all necessary documents to perform a comprehensive evaluation of the statements.

### 2.7. Advisory Board

The advisory board was composed of four medical experts and evaluated the presented statements. The experts provided detailed feedback, expressing their consent or dissent on each statement. In addition to indicating whether they agreed or disagreed, the experts assigned a confidence level ranging from 0 to 100, where 100 indicated absolute certainty and 0 an absolute uncertainty. Furthermore, they provided textual feedback explaining the reasons behind their judgment and any supporting evidence, including scientific publications, through a direct connection with PUBMED, allowing for an in-depth understanding of their positions.

**Figure 3:**
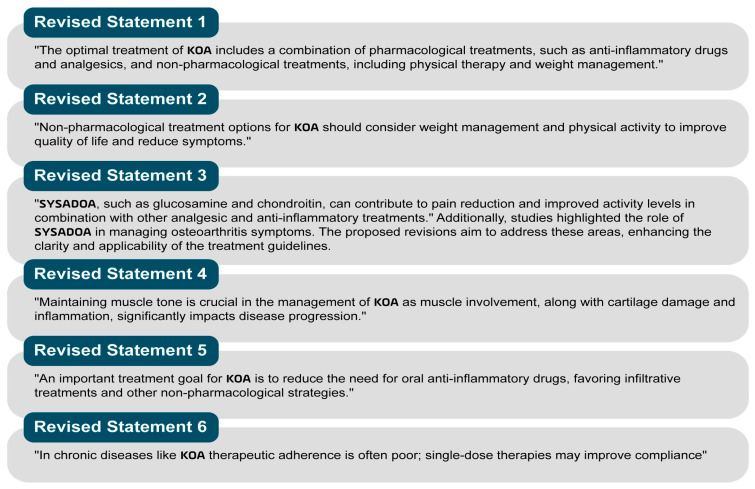
Revised statements.

The revised statements are shown in Figure 3.

The reformulation of the statements was assisted by AI during several sessions of advisory board meetings until they reached a consensus; this process also amended some accidental “hallucinations” that the LLM modeled which were not specifically necessary for medical purposes.


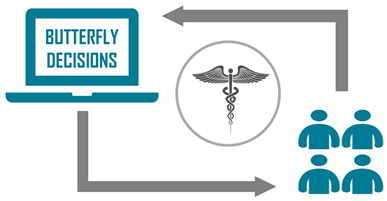


### 2.8. Consensus Analysis Report

A consensus analysis report has been produced to summarize the experts’ positions. In this report, two main indices were calculated: the Certainty Index and the Contradiction Index. The Certainty Index measures the overall confidence of the experts in the statements, while the Contradiction Index evaluates the level of disagreement among the experts. The combination of these indices determined whether a statement needs to be revised. Neither the first index nor the latter one alone are considered predictive tools of the efficiency of the software.

## 3. Results

Utilizing a software platform, board members evaluated all statements, providing their agreement levels, confidence, and supporting evidence. Based on these evaluations, a degree of certainty and contradiction was calculated for each statement.

**Figure 4:**
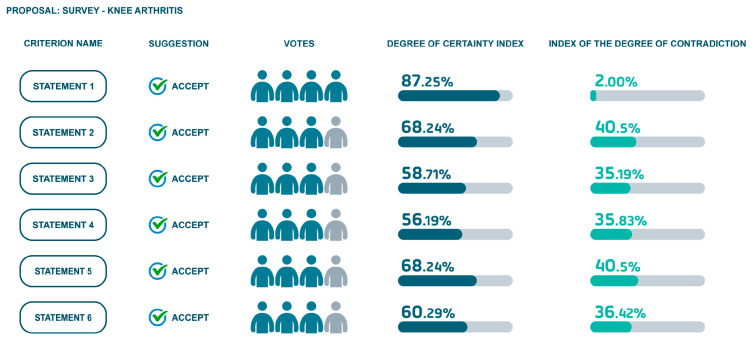
Results of statements’ agreement level in first AI session.

**Figure 5:**
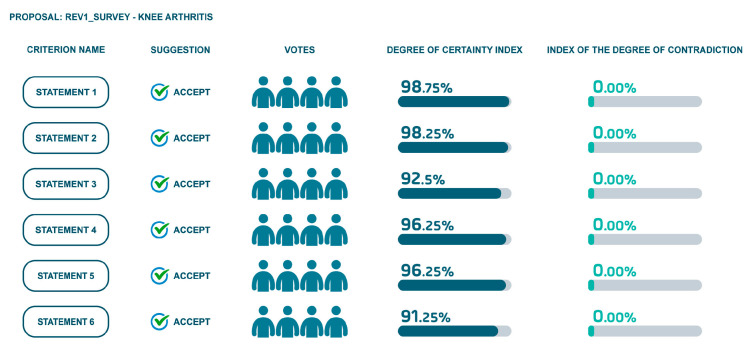
Results of revised statements’ agreement level.

The first session was conducted individually to evaluate statements, providing feedback and incorporating evidence. A second round of evaluation was conducted with all of the experts together to refine the statements and to achieve a higher consensus level. The results are shown in Figure 4 and Figure 5.

**Statement 1**: “The optimal treatment of KOA involves a combination of pharmacological and non-pharmacological treatments”.

Agreement Level: High (87.25% certainty, 2.83% contradiction).

Feedback Summary: Emphasized the inclusion of physical therapy and weight management.

**Revised Statement 1**: “The optimal treatment of KOA includes a combination of pharmacological treatments, such as anti-inflammatory drugs and analgesics, and non-pharmacological treatments, including physical therapy and weight management”.

**Statement 2**: “Non-pharmacological treatment of KOA must include weight loss and physical activity”.

Agreement Level: Mixed (68.24% certainty, 40.50% contradiction).

Feedback Summary: Disagreement on the mandatory inclusion of both elements.

**Revised Statement 2**: “Non-pharmacological treatment options for KOA should consider weight management and physical activity to improve quality of life and reduce symptoms”.

**Statement 3:** “Symptomatic Slow Acting Drugs for Osteoarthritis (SYSADOA) play an important role in reducing pain symptoms and improving activity levels”.

Agreement Level: Mixed (58.71% certainty, 35.19% contradiction).

Feedback Summary: Evidence supports the role of SYSADOA in combination with other treatments.

**Revised Statement 3**: “SYSADOA, such as glucosamine and chondroitin, can contribute to pain reduction and improved activity levels in combination with other analgesic and anti-inflammatory treatments”. Additionally, studies highlighted the role of SYSADOA in managing osteoarthritis symptoms. The proposed revisions aim to address these areas, enhancing the clarity and applicability of the treatment guidelines.

**Statement 4**: “Muscle involvement is as important as cartilage damage and inflammation in the genesis and progression of the disease”.

Agreement Level: Moderate (56.19% certainty, 35.83% contradiction).

Feedback Summary: Highlighted the importance of maintaining muscle tone.

**Revised Statement 4:** “Maintaining muscle tone is crucial in the management of KOA as muscle involvement, along with cartilage damage and inflammation, significantly impacts disease progression”.

**Statement 5:** “A key outcome of treatment is the reduction of oral anti-inflammatory drug consumption”.

Agreement Level: High (68.24% certainty, 40.50% contradiction).

Feedback Summary: Discussion on the role of infiltrative treatments.

**Revised Statement 5:** “An important treatment goal for KOA is to reduce the need for oral anti-inflammatory drugs, favoring infiltrative treatments and other non-pharmacological strategies”.

**Statement 6:** “In chronic diseases, poor therapeutic adherence is common; single-dose therapies, such as a food supplement with glucosamine sulphate 845 mg, chondroitin 500 mg, L-carnitine 500 mg, collagen 300 mg, bromelain 100 mg and vitamins D 25 µg have shown optimal compliance”.

Agreement Level: Mixed (60.29% certainty, 36.42% contradiction).

Feedback Summary: Some participants unfamiliar with the drug.

**Revised Statement 6**: “In chronic diseases like KOA therapeutic adherence is often poor; single-dose therapies may improve compliance, as seen with glucosamine sulphate 845 mg, chondroitin 500 mg, L-carnitine 500 mg, collagen 300 mg, bromelain 100 mg and vitamins D 25 µg supplementation”.

## 4. Discussion

AI is a useful tool for clinical decision support systems which can organize and analyze large amounts of medical data, allowing physicians to quickly access crucial information for patient care. AI is designed to act in a supportive role in clinical settings and can suggest treatment options based on a huge amount of scientific evidence and clinical data available, enabling physicians to make more informed and personalized decisions. AI and experts could work together to enhance diagnosis, treatment, and disease management, ensuring more effective and patient-centered care [25]. Although most doctors recognize the potential benefits of AI in healthcare, some maintain a cautious stance toward its adoption [26]. Increased confidence in AI tools, and understanding the technology, its potential benefits, and its limitations, are crucial for building trust. It is essential to develop competencies relating to AI, and recently, some universities have encouraged staff members and students to start using AI in daily work and studies [27,28,29]; Stanford University has even released the “AI teaching guide” [30].

As already mentioned, the NLP developed by AI has experienced explosive growth and NLP will probably revolutionize medical education in the future [31]. NLP can help medical students to memorize and understand a large amount of information, enabling them to participate in interactive learning experiences [32].

A recent work evaluated the reproducibility and reliability of ChatGPT’s responses to 19 statements regarding the management of hip fractures in older adults, as adopted by the American Academy of Orthopaedic Surgeons; the results showed that ChatGPT may be useful in providing guidelines for hip fractures, but performs poorly in terms of accuracy and precision [33]. In a clinical setting, a proper clinical validation of AI technologies is absolutely required.

The aim of this study was to integrate AI applications in clinical workflows and this is the first study that evaluated the incorporation of AI-assisted decision-making processes in KOA management. The consensus process was driven by Butterfly Decisions, a new software platform; after a survey based on the observations and comments of 30 physicians in 30 cases of KOA that were conservatively managed, four expert orthopedic surgeons evaluated six statements, extracted and summarized by AI software, and provided their levels of agreement with the statements.

Butterfly Decisions proved to be easy to use and each statement was structured to be clear, concise, and easily understandable by the board members.

Consensus was reached very quickly, and the results revealed a high level of agreement in KOA conservative management after only two board sessions; these good results were obtained for several reasons: a lot of literature and guidelines about the topic, the excellent analytical capabilities of AI, and exemplary cooperation between board members.

AI impact on doctor-to-doctor relationships has unexpectedly shown a positive effect, increasing agreement and decreasing dispute. AI improved collaboration between the four board members with varying levels of experience; in addition, this approach provides significant advantages, including a faster process and improved time management through remote tools. It is possible to assume that AI and language models could improve communication and collaboration between all healthcare professionals.

The Butterfly Decisions platform was able to analyze and process different human languages; in this study, data in two different languages were entered: Italian and English. In this way, it has been possible to successfully compare the data that were entered.

Despite these benefits, there are some important considerations and limitations: this study is a pioneering work and constrained by its small sample size of physicians; secondarily, the initial survey was presented without a validated questionnaire that was adapted from common clinical practice, developed by doctors rather than AI experts. Moreover, no diagnostic imaging was provided for the 30 cases. Finally, some hallucinations arose from the initial survey, requiring adjustments by the advisory board.

Regarding the method, the models used were not specifically trained for medical purposes and the assigned task.

Some “hallucinations” (unsupported statements) were observed: one of the clearer mistakes was the misinterpretation of the word “gonarthrosis” that AI initially evaluated as “gonorrhoea”. Thus, the word knee osteoarthritis was adopted to avoid this error. These “scientific accidents” may be easily mitigated in the future by more advanced models, targeted training, and through refining prompt engineering techniques.

Consequently, AI-assisted decision-making was proved to have excellent analytical capabilities, but algorithms need to be trained by orthopaedic experts with the correct inputs in order to mitigate risks associated with hallucinations. Thus, in our opinion, AI should be a human-centric technology.

## 5. Conclusions

In this study, an expert consensus analysis about knee osteoarthritis conservative management was facilitated by AI. The use of algorithms accelerates consensus analysis and reduces medical writing times, yet it remains essential for domain experts to supervise AI-generated results. There is a chance that language models may provide inaccurate or irrelevant information and so AI tools are most effective when combined with expert-led discussion sessions that can review and validate the outputs. In the future, additional efforts will be required to evaluate the incorporation of AI in clinical workflows.

## 6. Patents

The workflow process, along with the schematic overview of the study protocol, as well as the proprietary algorithms and software employed, are the intellectual property of Butterfly Decisions.

## Data Availability

Data reported on the manuscript.
